# Constrained metal-on-metal hip arthroplasty: ever heard of a 50-year survival story?

**DOI:** 10.1007/s00132-023-04380-8

**Published:** 2023-05-12

**Authors:** Andrzej Jasina, Andreas Enz, Annalena Knoop, Gareth Jones, Martin Ellenrieder, Wolfram Mittelmeier, Christoph Lutter

**Affiliations:** 1 Department for Orthopaedics, Rostock University Medical Center, Doberaner Str. 142, 18057 Rostock, Germany; 2 Leeds Beckett University, Leeds, UK

**Keywords:** Hip replacement, Sivash prosthesis, Prosthesis survival, Metal debris, History of arthroplasty, Hüftendoprothetik, Sivash-Prothese, Überlebenszeit von Endoprothesen, Metallabrieb, Geschichte der Endoprothetik

## Abstract

**Background:**

The history of total hip arthroplasty dates back to the first half of the twentieth century. Data on hip endoprostheses implanted during the 1960s and 1970s suggest widely varying survival rates of the prosthesis.

**Case:**

A case of a patient who underwent total hip arthroplasty in 1972 using a Sivash prosthesis, developed in 1956 in the former Soviet Union, is presented in this article. The prosthesis has remained unrevised in the patient’s body for 50 years and he continues to be widely free of implant-related symptoms. Despite the constrained metal-on-metal design of the implant, which can lead to adverse reactions to metal debris, no elevated systemic metal ion levels were detected.

**Conclusion:**

The likelihood of encountering patients with prosthesis survival beyond 50 years is still rare. Nevertheless, changing demographics and the steadily improving designs and materials of hip endoprostheses may likely result in such cases.

## Abstract (de)

**Hintergrund:**

Die Geschichte der Hüftendoprothetik reicht bis in die erste Hälfte des 20. Jahrhunderts zurück. Die Angaben zu Hüftendoprothesen, die in den 1960er- und 1970er-Jahren implantiert wurden, deuten auf sehr heterogene Überlebensraten der Prothesen hin.

**Der Fall:**

In diesem Beitrag wird der Fall eines Patienten vorgestellt, bei dem im Jahr 1972 eine 1956 in der ehemaligen Sowjetunion entwickelte Sivash-Prothese implantiert wurde. Die Prothese ist seit 50 Jahren unverändert im Körper des Patienten eingebaut, und er ist weiterhin weitgehend frei von implantatbedingten Symptomen. Trotz des Metall-auf-Metall-Designs des Implantats – das zu unerwünschten Reaktionen auf Metallabrieb führen kann – wurden keine erhöhten systemischen Metallionenspiegel festgestellt.

**Schlussfolgerung:**

Die Wahrscheinlichkeit, dass Patienten mit Prothesen, die länger als 50 Jahre erhalten bleiben, anzutreffen sind, ist immer noch gering. Dennoch können die sich verändernde Demografie und die sich ständig verbessernden Designs und Materialeigenschaften von Hüftendoprothesen dazu führen, dass solche Fälle auftreten.

## Introduction

Total hip arthroplasty (THA) is one of the most common surgical procedures worldwide [1]. In 2012, worldwide systematic comparative analysis revealed the annual number of THAs per 100,000 residents reached approximately 133 on average [2].

The history of joint replacement of traumatic and degenerative hip diseases has its beginnings much earlier than the last quarter of twentieth century, with the first reports dating back as far as eighteenth century [3]. Those first attempts, described by Park, mainly focused on the excision of the affected hip (cited from reference [3]). The era of THA began in the first half of the twentieth century. Moore implanted a stem in 1940, that became the first widely distributed product for femoral fractures. Work of McKee and Farrar, including the use of the acrylic cement, was a reference standard for many years to come [4]. The introduction of the low-friction arthroplasty by Charnley using a 22.25 mm head heralded a significant change to clinical procedures (cited from reference [5]). Nevertheless, the history of THA is not only the history of the techniques and implant designs but the history of materials and their pairing. Over the years materials like ivory, glass, teflon or vitallium have been proposed for use in hip arthroplasty [7, 8]. McKee and Farrar worked with cemented metal-on-metal implants. Charnley, however, switched to a metal design combined with a high-density polyethylene cup [4]. Recent approaches focused on further improvement of polyethylene designs, such as stabilization with vitamin E [3].

**Fig. 1:**
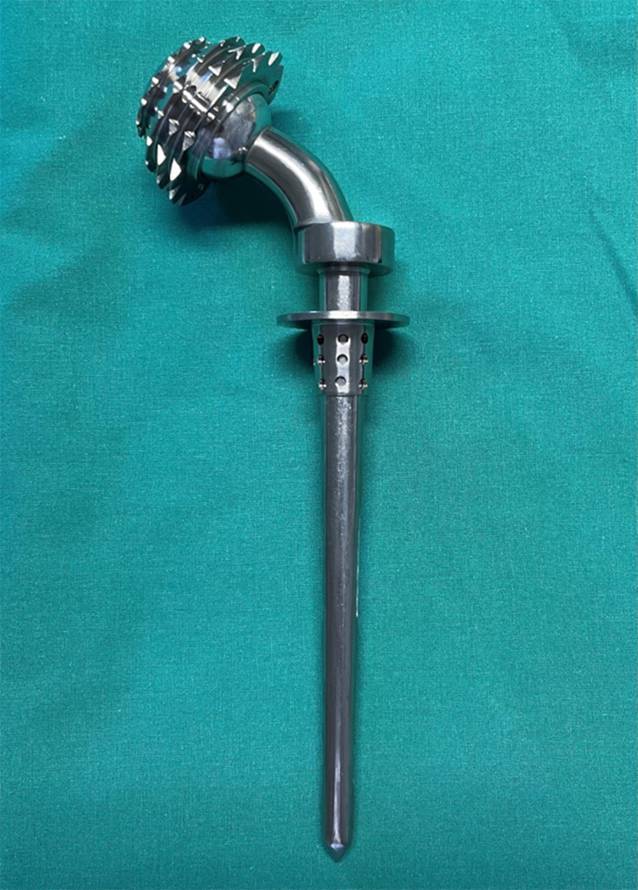
A Sivash metal-on-metal prosthesis, developed by Prof. Konstantin M. Sivash in 1956

Isolated by the political circumstances, Prof. Konstantin M. Sivash developed another prosthesis design on the eastern side of the iron curtain in 1956 which was introduced to the public in Moscow 7 years later [5]. His THA was a metal-on-metal implant with cobalt-chromium head and acetabular liner with titanium shell and stem (Fig. 1; [5]). It was designed as a monoblock-constrained device with the head permanently fixed to the stem neck and a constraining ring [5].

## Case presentation

**Fig. 2:**
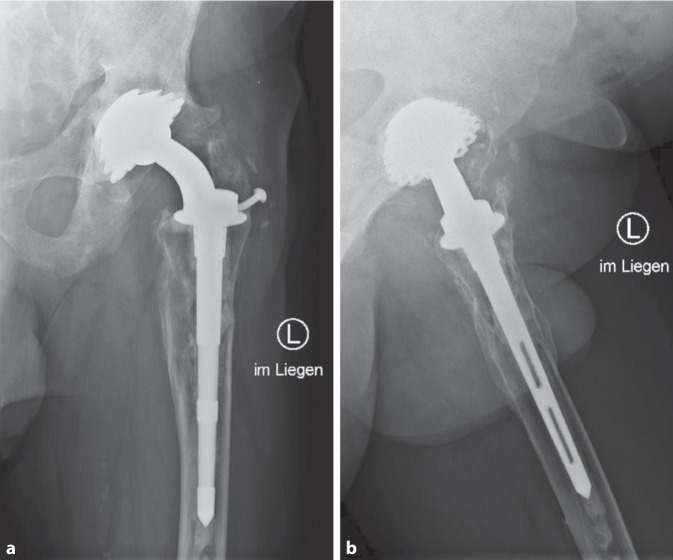
Anterior-posterior (**a**) and Lauenstein view (**b**) radiographs of the presented case (left hip). The left hip shows some periarticular ossifications. A screw that was inserted in the collar of the prosthesis has loosened over the years but appears to be locked in its current position. The patient has no complaints here. It is unclear whether some kind of cement was used during implantation of the prosthesis or whether the dense areas within the bone originate from the first operation

The 75-year-old male patient suffered a trauma at the age of 20 years and all procedures were undertaken in the former German Democratic Republic. He fractured his pelvis, the femoral neck on the left side, and suffered a concussion. The fracture was initially treated with an osteosynthesis. The surgery failed creating a 5 cm leg shortening on the left side and distinct gluteal insufficiency with positive Trendelenburg sign. In 1971 he underwent a Smith-Petersen mold cap and subsequently a cementless total hip arthroplasty (1972). Leg shortening was successfully reduced to 1.5 cm but muscle atrophy, movement restriction of the left hip and painful flexion of the left knee initially remained. Range of motion has been recorded twice over the years: in 2011 extension/flexion 0°/0°/90°, abduction/adduction 15°/0°/35°, external/internal rotation 60°/0°/0° and in 2020 extension/flexion 0°/5°/75°, abduction/adduction 20°/0°/20°, external/internal rotation 40°/0°/0°. Further slight increase of muscle atrophy of the left leg was found as well as mild pain with higher loads and increasingly unsteady gait. No use of gait support is needed up to date. Of note, the patient reports having performed high intensity sporting activities throughout these years such as jogging and hiking. The patient visited our outpatient clinic in November 2021 after having read in the media that our research team focuses on metal ion release from total joints. During the consultation he reported no current medical treatment. He reported an ongoing active lifestyle and continues to be employed. No daily intake of any medication was reported. The patient still walked without any aids, was able to climb and descend stairs safely. Further physical examination was unremarkable. Radiographs of the pelvis and the left hip showed a firm and correct fit of the hip prosthesis (Fig. 2).

The extremely long survival of the prosthesis combined with the specific metal-on-metal pairing of the prosthesis head and acetabular component raised questions about potential extensive metal abrasion within the joint. Therefore, we investigated levels of systemic metal ions in the patient’s whole blood (Table 1). Levels of aluminium (Al), copper (Cu), zinc (Zn), lead (Pb), molybdenum (Mo), nickel (Ni) and titanium (Ti) were within normal ranges. Chrome (Cr) and cobalt (Co) levels were found to be minimally elevated with 1.40 µg/l and 1.00 µg/l, respectively, but were not considered clinically significant. The patient was advised to continue his active lifestyle and to attend for an annual check-up.

**Table 1:** Metal ion levels in the patient’s blood

Element	Patient result	Standard range	Unit
Iron	16.5	8.1–32.6	µmol/l
Aluminium	< 5	< 10	µg/l
Copper (total)	828	560–1110	µg/l
Zinc	12.8	9.18–18.3	µmol/l
Chrome	**1.40**	< 0.4	µg/l
Cobalt	**1.00**	< 0.4	µg/l
Molybdenum	0.500	0.3–1.2	µg/l
Nickel	< 1.00	< 2.8	µg/l
Titanium	< 2.00	< 7.7	µg/l

Bold values are slightly elevated

## Discussion

The patient contacted our orthopedic department after reading a report on current research on metal ion release from total joint arthroplasties. Notably contact was initiated by pure interest in the subject area and not symptoms or suffering. To date, there is a paucity of literature on survival rates of the Sivash prosthesis. A serious problem limiting accurate scientific research is the fact that the vast majority of sources and studies concerning this model of prothesis were published in Russian. Many articles also do not have available source material or abstracts. In a case series from 1996, Schmalzried et al. reported a 46-year-old female patient, who underwent a THA with a Sivash prosthesis and did not experience any clinically relevant issues until 8 months prior to the revision surgery in 1993 (20 years survival) [6]. In 1980, Dobbs estimated the survival of a metal-on-metal THA to be 53% at 11 years [7]. The Sivash prosthesis was not among those listed in this article, with various of Stanmore prosthesis being investigated in his work. Dobbs also reported a better survival of the metal-on-plastic prosthesis in comparison with metal-on-metal implants [7].

Metal as a component material of hip prostheses was supposed to be the answer to the problems encountered with polyethylene [8]. Particles released by the wear of polyethylene were associated with one of the common causes of revision and failure of hip endoprostheses, aseptic loosening due to osteolysis [8]. The metal surfaces showed less annual wear than polyethylene [8]. In addition, the rigidity of the metal components in comparison with brittle ceramic parts enabled the use of thinner acetabular inlays, resulting in the use of heads with larger diameters, providing greater joint stability [8]. Furthermore, some data suggest lower long-term mortality in patients undergoing metal-on-metal resurfacing compared with patients undergoing cemented or uncemented total hip replacement [9]. It should be noted, however, that a direct comparison of hip resurfacing and total hip arthroplasty may be biased due to confounding factors, as discussed by the authors [9]. Despite its promising properties, while the wear issues of polyethylene have been largely resolved with the introduction of a cross-linked version [8], follow-up studies of metal-on-metal endoprostheses have shown its inferiority to prostheses that utilize other materials [10]. The main cause of complications and failures of metal-to-metal designs turned out to be metal ions; produced as the bearings of the prosthesis wore down [8]. Of the systemically detectable ions, such as cobalt, chromium, nickel, titanium or molybdenum, two (chromium and nickel) have been classified by the International Agency for Research on Cancer as “carcinogenic to humans,” and another (cobalt) as “probably carcinogenic to humans” [10]. The trend to depart from metal-on-metal pairing in Germany is clear. In the report of the German Arthroplasty Registry (EPRD) for the year 2020 the metal-on-metal pairing represented only around 0.2% of all registered THAs [11].

The case of the 46-year-old patient described by Schmalzried et al. seems to confirm that the Sivash prosthesis, which is a metal-to-metal design, was also not free from the problem of metal ion release. Analyses of the removed prosthesis showed wear of the titanium femoral neck of almost 50%, probably due to impingement and corrosion marks in the acetabulum [6]. Histological examination of tissues from around the components showed extensive fibrosis with evidence of macrophages containing large amounts of metal particles [6]. Considering the current data on the wear of metal surfaces with resultant production of metal ions, as well as the supporting clinical case reports from that period, it seems all the more surprising that the results of our patient’s blood tests did not show an elevated systemic concentration of metal ions.

Besides the material composition, the fixation technique also needs to be considered. In their meta-analysis of studies comparing cemented and cementless THAs, Abdulkarim et al. showed no difference in implant survival measured by revision rates [12]. Implantation of a Sivash prosthesis was performed via the direct lateral approach [5]. Uniquely, this involved osteotomy and reattachment of the greater trochanter with attached gluteal muscles to the prosthesis via a metal dowel inserted into the femoral component [5]. Preparation of the femoral canal was performed with cone cutters [5]. The fixation of the cup was ensured by press-fit effect and special blades of the prosthesis cup, pressed into the cup by hammering [5]. The Sivash prosthesis was designed as a constrained monoblock prosthesis. This design was intended to decrease the luxation rate, which seemed to be a problem especially with the then very popular low-friction design by Charnley [4]; however, the constrained approach caused a problem with femoroacetabular impingement, as highlighted in the case report by Schmalzried et al. [6]. In addition, given the constrained design, potential revision of the prosthesis required removal of the entire implant [13]. Further potential problems with the designed Sivash were indirectly highlighted by the United States Surgical Corporation, which in the 1970s purchased a license to manufacture the Sivash prosthesis. They modified the design using a 3° Morse taper and improved the stem construction to prevent femoral splitting and stem rotation [5]. None of those potential failure causes of the implant were observed in radiographic imaging of our patient (Fig. 2).

In the 1970s, state of the art designs included the McKee-Farrar prosthesis and the low friction Charnely prosthesis [4]. Long-term survival for both designs was reported in literature as 73–81% at the 20–25 years benchmarks [8]. Therefore the 50-years survival of our patient’s prosthesis is astonishing. The successor to the Sivash prosthesis was its modified version, the Sivash Range of Motion (S-ROM, DePuy Orthopaedics Inc., Warsaw, IN, USA), which was produced until 2007 [5].

Very long satisfactory results of joint replacement appear as confirmation of the efforts and successful research of the last 100 years. Many of the Sivash properties, such as the metal-on-metal bearing couple, the constrained design, the polished stem and the unusual macrostructure of the acetabular component have now been largely abandoned. The presented case seems to show that these prostheses can have extremely long survival times, and this without significant elevation of metal levels. The pioneering work of our predecessors around the world therefore cannot be credited highly enough; the early generations of arthroplasty laid the foundation for immense improvements in the quality of life for millions of people.

## Abbreviations

EPRD: German Arthroplasty Registry
S-ROM: Sivash Range of Motion
THA: Total hip arthroplasty

## Declarations

For this article no studies with human participants or animals were performed by any of the authors. All studies mentioned were in accordance with the ethical standards indicated in each case. For images or other information within the manuscript which identify patients, consent was obtained from them.
